# The Value and Dangers of "A.C.E." and "C.E." Mixtures

**Published:** 1909-07-24

**Authors:** 


### Anæsthetics.

### THE VALUE AND DANGERS OF "A.C.E." AND "C.E." MIXTURES.

Mixtures of alcohol, chloroform, and ether in various proportions have been extensively employed with the idea of obtaining an anæsthesia free from the inconveniences of ether and from the dangers of chloroform; and opinions of the most contradictory character have been expressed in regard to their use in practice. Not many years ago a well-known surgeon, at a discussion at a medical society, deliberately denounced A.C.E. as a "damnable mixture," whilst another extolled its value in all perilous cases. In this article an endeavour will be made to show that, as in most hotly debated questions, "both are right and both are wrong."

As a mixture is often selected for administration to patients in a condition unfavourable for any anæsthetic—thought to be unfit for either ether or chloroform alone—it is not surprising that some may be prejudiced by unfortunate experiences.

The chief theoretical objection usually stated is that in any mixture containing ether and chloroform the former evaporates before the latter, so that the anæsthetist, thinking himself safe, is apt to give an overdose of chloroform. In fact, as it has been said, that "Nobody knows what is being given."

Now laboratory experiments have indicated that in A.C.E., when carefully prepared, we have a true chemical compound, not a mere mechanical mixture, and that its vapour is not merely that of its separate components. Most authorities, however, regard the addition of alcohol as unnecessary, and use a C.E. mixture (chloroform 2, ether 3). Be this as it may, the objection may be obviated by proper precautions.

Nothing in the shape of a closed inhaler should be employed. With children, weakly or nervous patients, the administration may well be begun, and even continued, with lint, or a mask, such as Schimmelbusch's, kept at a short distance from the face. With others, a "Rendle's," or similar mask, should be used. This should not, as a rule, be closely applied, and should be removed altogether at frequent intervals. The air holes should not be obstructed, and the sponge should not be of very fine texture nor tightly packed. It should not be saturated at long intervals with half an ounce or more of the fluid. Small quantities should be dropped on it frequently, ensuring an almost continuous supply of fresh mixture. In long operations a mixture may well be given from a Junker's inhaler, the same care being taken to replenish the bottle frequently with small quantities.

The patient's breathing, colour, pupils, and pulse must receive the same unremitting attention as in giving chloroform. An overdose is indicated mainly by failure of respiration and over-dilatation of the pupils. Unless due to a sudden overdose (which should not occur), there is, however, in most cases quite sufficient warning to a watchful administrator. On the other hand, the signs of danger are sometimes so insidious that an inexperienced or careless administrator may find himself unexpectedly in a position of some anxiety. If the breathing tend to become shallow or hesitating, if at the same time the corneal reflex be absent, the pupils dilated and sluggish, and the muscles flaccid, the mask should be at once withdrawn, the lips and cheeks briskly rubbed, and traction made upon the tongue. Fortunately, even when respiratory movements cease, there is not that depression of the circulation which occurs under chloroform, and they are generally speedily resumed on artificial respiration being performed.

Neither this anæsthetic nor any other should be given indiscriminately as a matter of routine. It is suitable more particularly in the following cases:

Weakly infants and old people, to whom it is not usually advisable to give ether. Robust and full-blooded young and middle-aged adults, according to the writer's experience, are generally anæsthetised more quietly and effectually by A.C.E. than by a gas-and-ether or ethyl-chloride-ether sequence, and more safely than by chloroform. This is especially the case in free-living alcoholic subjects.

As noticed in previous articles, there are many operations in which ether is apt to cause difficulties to the surgeon and to interfere with the success of the proceedings and with the ultimate welfare of the patient. C.E. or chloroform is then preferable, and if the administrator be inexperienced the former should be chosen, particularly if the patient be feeble or the operation likely to be long, or to cause reflex functional disturbances.

C.E. is the best anæsthetic in most cases of respiratory difficulty, heart disease, and obesity, when nitrous oxide or ether are often dangerous. It may be used as a preliminary to ether for nervous patients, who make much objection to a closely fitting face-piece.

To sum up, the writer considers it an anæsthetic valuable as regards both convenience and safety; but only when properly given to a properly selected case.

